# Triblock Superabsorbent Polymer Nanocomposites with Enhanced Water Retention Capacities and Rheological Characteristics

**DOI:** 10.1021/acsomega.1c06961

**Published:** 2022-06-08

**Authors:** Yeşim Menceloğlu, Yusuf Ziya Menceloğlu, Senem Avaz Seven

**Affiliations:** † Faculty of Engineering and Natural Sciences, Sabanci University, Tuzla, 34956 Istanbul, Turkey; ‡ Sabanci University Integrated Manufacturing Technologies Research and Application Center & Composite Technologies Center of Excellence, Teknopark, Pendik, 34906 Istanbul, Turkey; § Sabanci University Nanotechnology Research and Application Center, SUNUM, 34956 Istanbul, Turkey

## Abstract

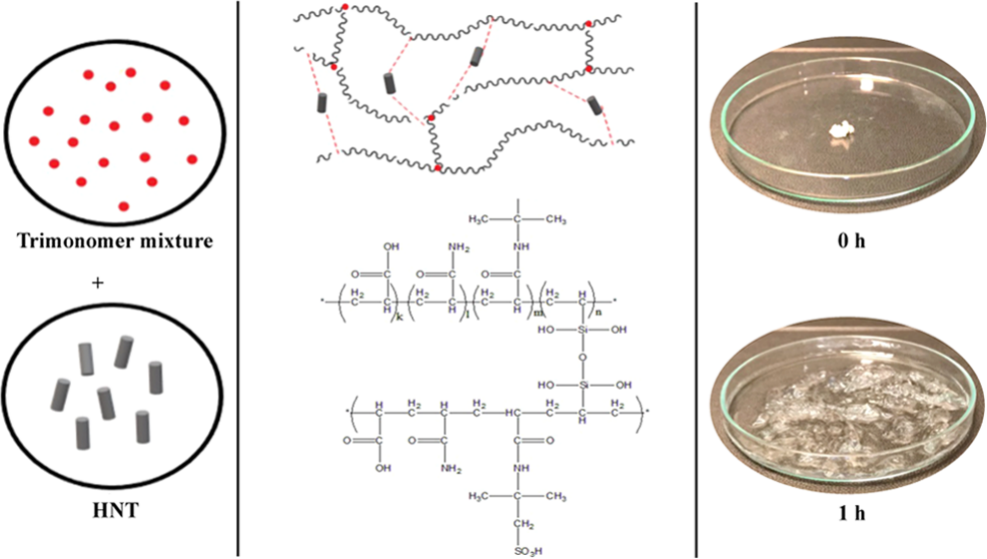

Superabsorbent polymers (SAPs) are useful polymers in a wide range of application fields ranging from the hygiene industry to construction and agriculture. As versatility and high water absorption capacity are their important merits, SAPs usually suffer from low water retention capacity (fast release) and weak mechanical properties. To address these drawbacks, a set of new superabsorbent polymer–Halloysite nanotube (HNT) nanocomposites was synthesized via free radical polymerization of acrylamide, 2-acrylamido-2-methylpropane-1-sulfonic acid, and acrylic acid in the presence of vinyltrimethoxysilane (VTMS) as the crosslinker. FTIR and TGA characterizations confirm the polymerization of SAP and successful incorporation of HNTs into the SAP polymer matrix. The effect of the HNT nanofiller amount in the nanocomposite polymer matrix was investigated with swelling–release performance tests, crosslink density calculations, and rheology measurements. It was found that equilibrium swelling ratios are correlated and therefore can be tuned via the crosslink densities of nanocomposites, while water retention capacities are governed by storage moduli. A maximum swelling of 537 g/g was observed when 5 wt % HNT was incorporated, in which the crosslink density is the lowest. Among the SAP nanocomposites prepared, the highest storage modulus was observed when 1 wt % of nanofiller was incorporated, which coincides with the nanocomposite with the longest water retention. The water release duration of SAPs was prolonged up to 27 days with 1% HNT addition in parallel with the achieved maximum storage modulus. Finally, three different incorporation mechanisms of the HNT nanofiller into the SAP nanocomposite structure were proposed and confirmed with rheology measurements. This study provides a rapid synthesis method for SAP nanocomposites with enhanced water retention capacities and explains the relationship between swelling and crosslink density and water retention and mechanical properties of SAP nanocomposites.

## 1 Introduction

Superabsorbent polymers (SAPs) are lightly crosslinked hydrogels with a three-dimensional structure possessing the ability to absorb and retain a high amount of water. They swell right after contact with water due to the differences in the osmotic pressure and the presence of hydrophilic functional groups such as -OH, -COOH, -CONH-, -CHO, -COONa, -SO_3_H, and -NH_2_ in the polymer backbone. The reason behind SAPs swelling instead of dissolution in aqueous medium is the chemical or physical crosslinking introduced into the polymer network structure via a crosslinker during or after the polymerization. This way, crosslinked SAPs can absorb a large amount of water and hold in their 3D structure.^1−3^

SAPs are classified as natural or synthetic with respect to their sources. Cellulose,^4^ saccharides,^5^ collagen,^6^ alginate,^7^ and chitosan^8^ are some examples of SAPs from natural sources.^9^ On the other hand, acrylamide,^10,11^ acrylic acid,^10,12^ methacrylic acid,^13^ hydroxyethyl methacrylate,^14^ poly(vinyl alcohol),^15^ and various acrylates are commonly used in the synthesis of synthetic SAPs. Recently, synthetic superabsorbent polymers have been more preferable than natural ones because of their higher absorption capacity,^16^ more durable gel life,^17^ and higher mechanical strength.^18^ In addition, combined SAPs can be prepared using both natural and synthetic polymers.^19^ These are generally synthesized via graft polymerization.^20^

SAPs are a special class of hydrogels with very high absorption capacities. While the swelling or absorption capacity of hydrogels is up to 10 g/g, SAPs are able to swell and absorb water up to 600–1000 g/g.^2−4^

Low elastic modulus, low fracture energies, and negligible fatigue resistance are the main problems associated with hydrogels since the energy cannot be dissipated effectively through the 3D polymer network. This results in the weak and brittle mechanical properties of hydrogels.^21,22^ In addition to that, the molecular weight between crosslinks is not uniform since the crosslinking points are placed disorderly in the polymer network. Therefore, the stress cannot be dissipated in the intrinsic structure, and microcracks form.^23^ Even though synthetic SAPs have relatively higher gel strength than that of natural SAPs, the strength of the gel is usually lost after reaching equilibrium swelling^24^ and usually disintegrated under load.^25^ To enhance the mechanical strength of SAPs, different approaches are used such as grafting with clays,^26^ interpenetrating network hydrogels,^27^ double-network hydrogels,^28^ nanocomposite hydrogels,^29^ or macromolecular microsphere hydrogels.^23^ The reasons behind the uniqueness and smartness of hydrogels are their characteristic response to external stimuli such as pH,^30,31^ ionic strength,^32^ solvent composition, temperature, and light.^2,33^ Hence, hydrogels are effective materials in a wide range of fields. Although 80% of the superabsorbent polymers are produced for personal care products such as diapers and sanitary pads, during the last years, they have been used in various application areas such as biomedical materials,^34^ food packing,^35^ drug delivery,^36^ adhesives,^37,38^ agriculture,^39^ and wastewater treatment.^40^ The high water absorption capacity, biocompatibility, softness, and sensitivity to environmental stimuli make SAPs preferable for a wide variety of applications.

The utilization of SAPs in agriculture has been coming into prominence day by day due to the increasing need of smart agricultural solutions. SAPs are mainly used as water reservoirs under soil for plants.^41^ The unique swelling and retaining characteristics of superabsorbent polymers make them suitable for agricultural applications. Water is one of the essential parameters in plant growth for the uptake of nutritive elements.^42^ On the other hand, global warming and climate change cause irregularities in the rainfall patterns, resulting in long-term drought in many agricultural lands in the world. Therefore, limited water sources should be used efficiently to overcome water shortage; however, the traditional manual irrigation systems in agriculture cause inefficient use of water, resulting in the contamination of natural resources and water loss. Today, 70% of freshwater in the world is consumed in agriculture, and this critical amount indicates that intelligent agricultural products will be an effective solution for sustainable agriculture by facilitating less water use.^43,44^ In this sense, SAPs provide smart solutions to overcome the indiscriminate consumption of water in agricultural irrigation.

Majority of the literature examples deal with the effect of SAPs on soil moisture, plant growth, and seedlings and indicate that the presence of SAPs near the plant roots increases the water absorption and retention capacity of soil, improving the plant quality.^45^ In addition, the irrigation frequency and excess water use are reduced by the utilization of hydrogels.^46^ Moreover, SAPs enable agriculture in areas under drought stress due to their water holding capacity.^47^ Once SAPs are placed under the soil near the roots, they fulfill the water demand of the plants by releasing water due to the osmatic pressure difference.^48^ Mixing superabsorbent polymers with soil increases the physical properties of the soil, plant yield, and seed germination.^49^

The polymer performance is enhanced by the introduction of triblock copolymers since they enable tailoring of the final properties according to application conditions.^32^ The most commonly used monomers for SAP synthesis are acrylic acid (AA) and its salts and acrylamide (AM).^50^ It has been reported that the stability of AM is increased with the addition of 2-acrylamido-2-methylpropane-1-sulfonic acid (AMPS).^51^ The presence of sulfonic acid groups in the copolymers of AM and AMPS increases the viscosity and stability of the main chain at high temperature and high salinity.^51^ Another study has shown that copolymerization of acrylamide with an ionic comonomer such as acrylic acid (AA) improves the swelling capacity and pH sensitivity of SAPs.^52^ The ability of swelling is increased with ionizable groups such as -COO^–^ and -SO_3_^–^ present in AA and AMPS comonomers.^53^ The presence of different comonomers such as AM, AA, and AMPS in a polymer chain provides tunability in swelling capacity, pH sensitivity,^53^ and mechanical properties.^54^

Nanocomposite hydrogel preparation is another approach to tune the swelling capacity and rheological characteristics of hydrogels.^55^ The mechanical^56^ and thermal^57^ properties and biocompatibility^58^ of hydrogels are increased via incorporation of clay with hydrogels. Attapulgite,^59^ montmorillonite,^60^ kaolin,^61^ and bentonite^62^ are commonly used clay minerals in the preparation of nanocomposite hydrogels. Halloysite natural nanoparticles are similar to kaolinite with the composition of aluminum oxide- and silicon oxide-based layers with a chemical formula of Al_4_Si_4_O_10_(OH)_8_·4H_2_O.^63^ Halloysite nanotubes (HNTs) have received considerable attention due to their high mechanical strength and modulus^64^ and large surface area and pore volume.^65^ In previous SAP research, it was shown that the compression strength, modulus, and toughness of reinforced hydrogels with HNTs are enhanced compared to neat hydrogels.^65,66^

One of the approaches in SAP synthesis includes the copolymerization/crosslinking technique, in which the crosslinker is inserted into the polymer backbone as a monomer. Also called as bulk crosslinkers, these agents are bifunctional or multifunctional monomers possessing one or more unsaturated bonds in their chemical structure and usually are chemically more active than other monomers.^67^ In that sense, alkoxysilanes, having both organic and inorganic functional groups, are characterized as multifunctional bulk crosslinkers, as they are able to form multiple covalent bonds between inorganic and/or organic species.^68,69^ They are advantageous in terms that they help establish covalent bonding between SAPs and HNTs to form a nanocomposite structure. In addition to this, the polymers/polyelectrolytes crosslinked by trialkoxysilane groups exhibit improved water durability.^70^

The present study explains the preparation novel SAP nanocomposites with improved swelling and water retention capacity and aims to comprehend and overcome the general problems associated with the weak mechanical properties of hydrogels. The synthesized SAP is a triblock random copolymer consisting of acrylic acid (AA), acrylamide (AM),, and 2-acrylamido-2-methylpropane-1-sulfonic acid (AMPS) monomeric units in the presence of the vinyltrimethoxysilane (VTMS) crosslinker.

## 2 Experimental Section

### 2.1 Materials

The monomers acrylamide (AM, ≥99% HPLC) and 2-acrylamido-2-methylpropane-1-sulfonic acid (AMPS, ≥99%) were purchased from Sigma-Aldrich and used without any further purification. Acrylic acid (AA, analytical grade) was purchased from Alfa Aesar and used as received. The initiator ammonium persulfate (APS, ≥99%) and the crosslinker vinyltrimethoxysilane (VMTS, 98%) were obtained from Sigma-Aldrich. Sodium hydroxide solution, 10 mol/L, and ethanol (EtOH, absolute-99.9%) were purchased from Merck and used without further purification. HNTs were kindly provided by ESAN. All solutions were prepared with distilled water.

### 2.2 Methods

#### 2.2.1. SAP Synthesis

SAPs were prepared via free radical polymerization in deionized water. The random copolymer was synthesized from acrylic acid (AA), acrylamide (AM), and 2-acrylamido-2-methylpropane-1-sulfonic acid (AMPS) monomers using ammonium persulfate (APS) as the free radical initiator, in the presence of vinyltrimethoxysilane (VTMS) as the crosslinking agent. Given amounts of AM and AMPS were dissolved in water, and then, AA was added to the solution mixture. After complete mixing took place and a clear solution was obtained, the highly acidic monomer solution, due to the acrylic acid, was neutralized using 12 M sodium hydroxide to adjust the pH to 8. Thereafter, the monomer solution was transferred into a three-necked flask connected to a condenser apparatus and purged with nitrogen to remove excess oxygen present in the reaction environment. After the mixture was bubbled with nitrogen gas for 15 min, VTMS was added to the mixture under vigorous mixing. The mixture was subjected to further mixing and bubbling for an extra 15 min to remove any excess oxygen and air from reaction medium. In another flask, the APS free radical initiator was dissolved in water and then added to the reaction mixture. The polymerization was carried out under reflux at 78 °C in an oil bath for 2 h. The amounts of monomers used in SAP synthesis are given in Table 1.

**Table 1:** Amounts of Monomers and HNTs Used in SAP and SAP-HNT Nanocomposite Synthesis

	AM (equiv mol)	AMPS (equiv mol)	AA (equiv mol)	VTMS (equiv mol)	HNT (wt %)
SAP2HNT0	0.06	0.005	0.07	0.001	0
SAP2HNT1	0.06	0.005	0.07	0.001	1
SAP2HNT5	0.06	0.005	0.07	0.001	5
SAP2HNT7	0.06	0.005	0.07	0.001	7
SAP2HNT9	0.06	0.005	0.07	0.001	9

A transparent viscous polymer solution was obtained at the end of the reaction. The three-necked flask was then taken out from the oil bath, and the polymer solution was cooled to room temperature. Finally, the resulting polymer was precipitated in ethanol in the form of white powder. The superabsorbent polymer was then dried at 70 °C for 2 days.

#### 2.2.2. Preparation of the SAP-HNT Nanocomposite

After the monomer solution was prepared and the pH was adjusted to 8 with 10 M NaOH, an amount of HNT was added to the monomer solution. The polymerization process was continued as in the SAP synthesis. First, VTMS was added to the inert reaction medium. The initiator was then added to the reaction mixture, and polymerization was carried out at 78 °C for 2 h. When the polymerization was finished, an opaque viscous polymer solution was obtained with precipitation in ethanol. The precipitated polymer powder was then dried at 70 °C or 2 days. The amounts of HNTs and monomers used in the SAP-HNT nanocomposite synthesis are given in Table 1.

### 2.3 SAP Characterization

#### 2.3.1. ATR/FTIR Spectroscopy

The synthesized SAP and SAP-HNT samples were characterized by attenuated total reflectance Fourier transformed infrared spectra (FTIR) collected on a Thermo Scientific iS10 ATR-FTIR spectrometer (Thermo Fisher Scientific) from 550 to 4000 cm^–1^, with a resolution of 0.5 cm^–1^. A total of 32 scans were gathered and baseline-corrected.

#### 2.3.2. Gel Permeation Chromatography (GPC)

The molecular weight of the noncrosslinked SAPs were determined using an OMNISEC gel permeation chromatograph (GPC, Malvern Panalytical, U.K.) equipped with a light scattering detector. The noncrosslinked polymer was dissolved in water at 35 °C. The flow rate was 0.7 mL/min, and the injected volume was 100 μL, with a sample concentration of 1 mg/mL. A triple-column system was employed, connected in series, Malvern CLM3000, Malvern CLM3003, and Malvern CLM3005, to determine the molecular weight ranges of 1, 3, and 5 k, respectively.

#### 2.3.3. Density Determination

The density values of SAP and SAP-HNT nanocomposites were determined sing an automatic gas pycnometer (Quantachrome Ultrapyc 1200e). Throughout the measurements, helium gas was purged at a flow rate of 1 mL/min for measurements.

#### 2.3.4. Rheometry

The rheological behavior of the synthesized polymers was characterized using an oscillatory rheometer (Anton Paar-Physica, MCR 702 TwinDrive, AUT). The rheological measurements were performed at 25 °C with a parallel plate (plate diameter of 25 mm, gap of 1 mm). For the rheology measurements, the synthesized polymers in the SAP2 dataset were swollen to maximum in distilled water for 24 h. A strain sweep test was performed to determine the test conditions in the linear viscoelastic (LVE) range. The shear strain value in the LVE range was determined at 6.2 Hz frequency and 0.01% amplitude gamma.

#### 2.3.5. Thermogravimetric Analysis (TGA)

The thermal behaviors of powder SAP and SAP-HNT samples were characterized by simultaneous thermal analysis (Netzsch STA, 449 C Jupiter, GER) using a differential thermogravimetric analyzer with a 50 mL/min flow from 30 to 1000 °C at a linear heating rate of 10 °C/min under a N_2_ atmosphere.

### 2.4 SAP Performance Test

#### 2.4.1. Swelling

The dried powder SAP and SAP-HNT nanocomposite samples are weighed before swelling in distilled water. The equilibrium swelling ratio (ESR) is calculated according to eq 1
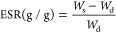
1where *W*_s_ is the weight of the hydrogel at equilibrium and *W*_d_ is the weight of the dry polymer. The water release ratios were calculated with the same equation (eq 1); only the ESR mark was changed to EShR to emphasize the contrast between swelling and release ratios.

#### 2.4.2. Determination of the Crosslinking Density

The ratios of crosslinked points of the hydrogels could not be determined with nuclear magnetic resonance (NMR) spectroscopy since the superabsorbent polymers were not dissolved in any solvent, only swollen in water. The number of crosslinked points is a determinant factor of the equilibrium swelling ratio values because the maximum swelling capability depends on the average molecular weight between crosslinked points. Therefore, determination of the crosslinking density through the average molecular weight between crosslinked points is required to clarify the swelling capacities of SAPs.

Hence, the crosslinking density and the average molecular weight between crosslinks were calculated by Flory–Rehner theory of swelling.^71^ The average molecular weight between crosslinks is calculated by the equation^72^
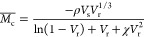
2where *V*_s_ and *V*_r_ denote the molar volume of the solvent (18 g cm^–3^) and volume fraction of the SAP in the swollen polymer, respectively, ρ stands for the density of dry SAP, and χ is the Flory–Huggins interaction parameter calculated from
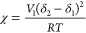
3Here, *V*_1_ denotes the molar volume of the solvent and δ_2_–δ_1_ states the Hildebrand solubility parameters (cal^1/2^ cm^–3/2^) for water and SAP, respectively. Once *M*_C_ is obtained from eq 2, the crosslinking density is calculated by
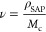


## 3 Results and Discussion

### 3.1 Structural Characterization

**Figure 1:**
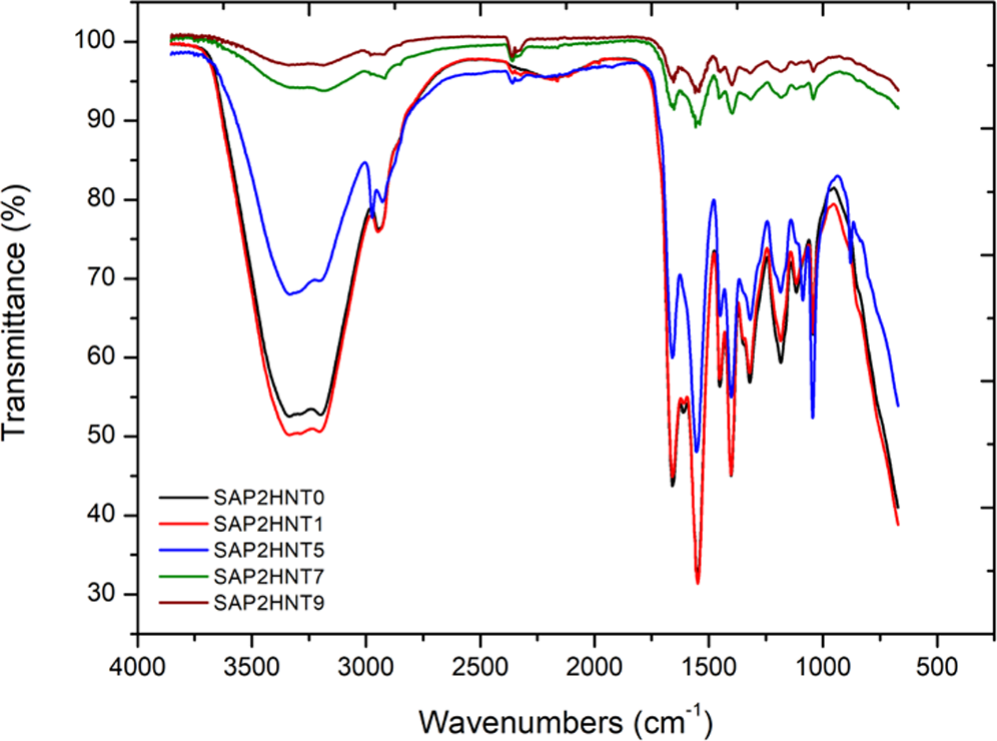
FTIR results of SAP2 polymers.

As SAPs are challenging to be characterized by NMR spectroscopy, the polymeric structure of SAPs was confirmed via FTIR analysis. The FTIR spectra of the synthesized SAP2 polymer set are demonstrated in Figure 1. FTIR confirms the presence of AM, AA, and AMPS repeat units and the VTMS crosslinker in the polymer backbone. In general, the FTIR bands appearing at 1190 and 1040 cm^–1^ correspond to the -Si-O- and Si-O-Si stretching vibrations in the VTMS structure, respectively. The broad peak between 3339 and 2985 cm^–1^ wavenumbers is attributed to the -OH vibrations in AM, AA, AMPS, and VTMS. The sharp FTIR band at 3339 cm^–1^ is due to the N-H stretching vibration of AM. The doublet peak at 2934 cm^–1^ is attributed to the stretching vibrations of -CH at the polymer backbone. The stretching vibrations of the primary and secondary amides -C=O in AM, AA, and AMPS structures are observed at 1600–1650 cm^–1^.^73^ The bands observed at 1447 and 1411 cm^–1^ are due to the stretching vibrations of -C-N and -NH_2_ in the acrylamide chemical structure, respectively. The peak at 1190 cm^–1^ corresponds to the sulfone groups present in the AMPS chemical structure. Additional bands observed in the SAP2HNT7and SAP2HNT9 FTIR spectra at 2360 cm^–1^ indicate the possibility of -CO_2_ absorption.

The FTIR spectra of the synthesized nanocomposites comprising HNTs are also presented in Figure 1. The Si-O- and Si-OH vibration bands observed at 1100–1050 cm^–1^ confirm the incorporation of HNTs in the polymer structure. According to FTIR results, polymerization was carried out with the participation of all monomers, and the presence of HNTs in the polymer structure was confirmed.

### 3.2 Thermal Characterization

**Figure 2:**
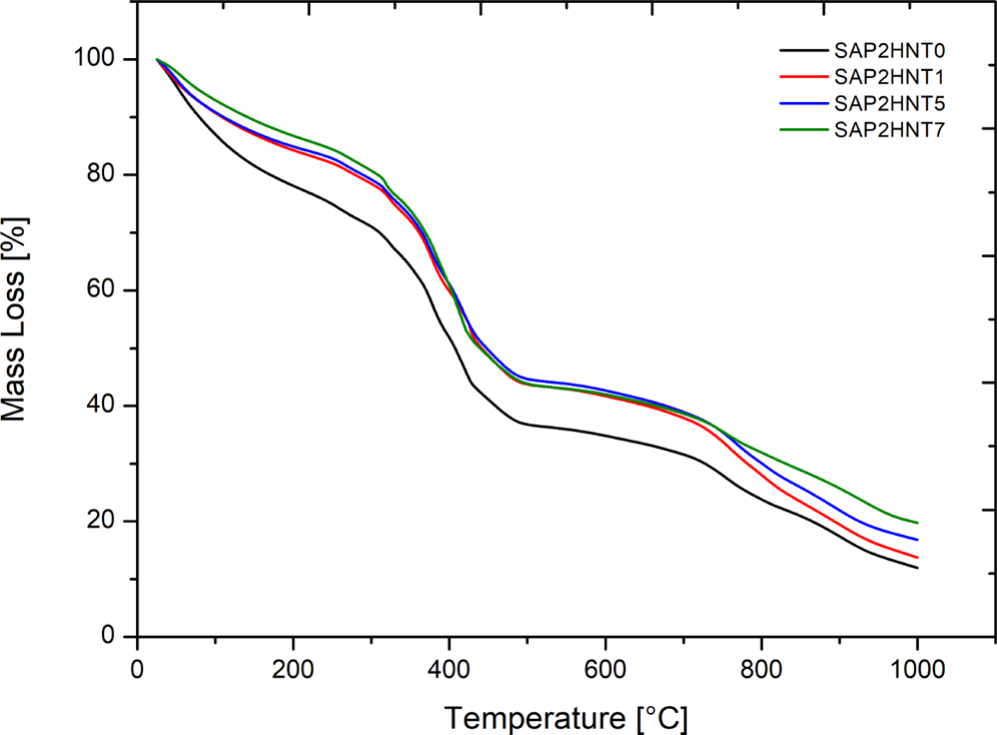
Thermogravimetric analysis of SAP and SAP-HNT nanocomposites.

The thermogravimetric analysis results of SAP and SAP-HNT nanocomposites are presented in Figure 2. The trio-random copolymer structure of SAPs and the addition of HNTs in 0, 1, 5, 7, and 9 wt % into SAP nanocomposite structures are verified by TGA results. In the thermograms (Figure 2), the first mass loss of 15% arising from the moisture content of the polymers is observed between 50 and 200 °C. In the second mass loss regime, AM and AMPS monomers within the polymer structure degrade at 200–300 °C. In the third regime, the sulfonic groups decompose above 300 °C. The residual mass values in nanocomposites containing 0, 1, 5, and 7% of HNTs are determined as 12, 14, 17, and 19% respectively, demonstrating a linear correlation between the HNT amount and the residual mass. TGA results confirm that the thermal properties of SAPs are improved with HNT addition and the residual mass of the polymer increased in direct proportion to the amount of HNTs found in the nanocomposite structure.

### 3.3 Molecular Weight and Density

The molecular weights of the SAPs were determined by GPC. To perform the analyses, SAP polymerization was carried out in the absence of the crosslinker, as polymers should be soluble in designated solvents for the GPC analysis. The molecular weights of the trio-random noncrosslinked polymers are presented in Table 2. The polydispersity index (PDI) value, Mw/Mn, is recorded as 1.276. This gives a broad distribution in the length of the polymer chains, which is expected since the SAP synthesis was carried out through free radical polymerization.

**Table 2:** Molecular Weight of the Noncrosslinked SAP and Density Values of SAP and SAP-HNT Nanocomposites

molecular weight of the SAP (noncrosslinked) (Da)		density of the SAP-HNT nanocomposite (g/cc)	
Mn-	940.9	SAP2HNT0	1.39
Mw-	1.200 × 10^6^	SAP2HNT1	1.61
Mz-	1.399 × 10^6^	SAP2HNT5	1.52
Mp-	1.419 × 10^6^	SAP2HNT7	1.69
Mw/Mn (PDI)	1.3	SAP2HNT9	1.72

We have further calculated the densities of the SAPs, as the density value is an input for the crosslinking density calculation. Densities of the SAP and SAP-HNT nanocomposites were measured using a gas pycnometer. Table 2 shows the densities of the synthesized SAPs. A foreseeable increase in the densities of SAP-HNTs is observed with respect to the HNT amount, except for SAP2HNT5. The reason for the difference in SAP2HNT5 could be the incomplete participation of HNTs in the nanocomposite structure.

### 3.4 Swelling Behavior of the SAPs

**Figure 3:**
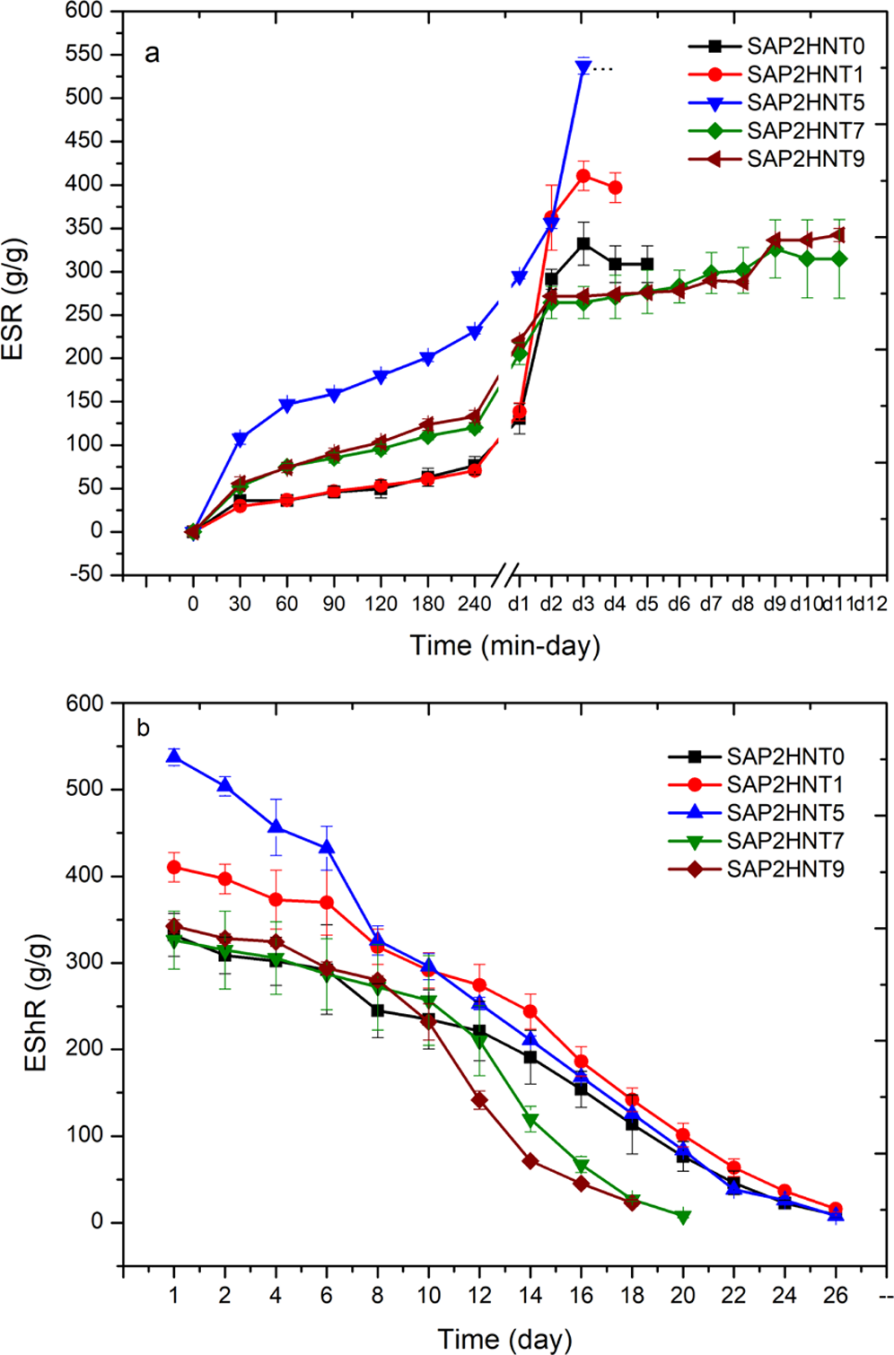
Swelling (a) and water release (b) test results of the SAP2 polymer set involving different amounts of HNTs.

Swelling tests are performed through the whole SAP dataset consisting of five different nanocomposites with varying HNT amounts (0, 1, 5, 7, and 9 wt %). Swelling tests demonstrate that (Figure 3a) the incorporation of 5% HNT provides the maximum swelling, while the swelling ratios decrease further with the HNT amounts. In line with this, Figure 3b presents that the slowest water release was obtained in 27 days with SAP2HNT1. In addition, these release trends after the 10th day also demonstrate that the SAP2HNT1 nanocomposite preserves the highest amount in its structure. In other words, incorporation of 1% HNT provides a better sustained water release trend compared to 5, 7, and 9% HNT addition.

**Figure 4:**
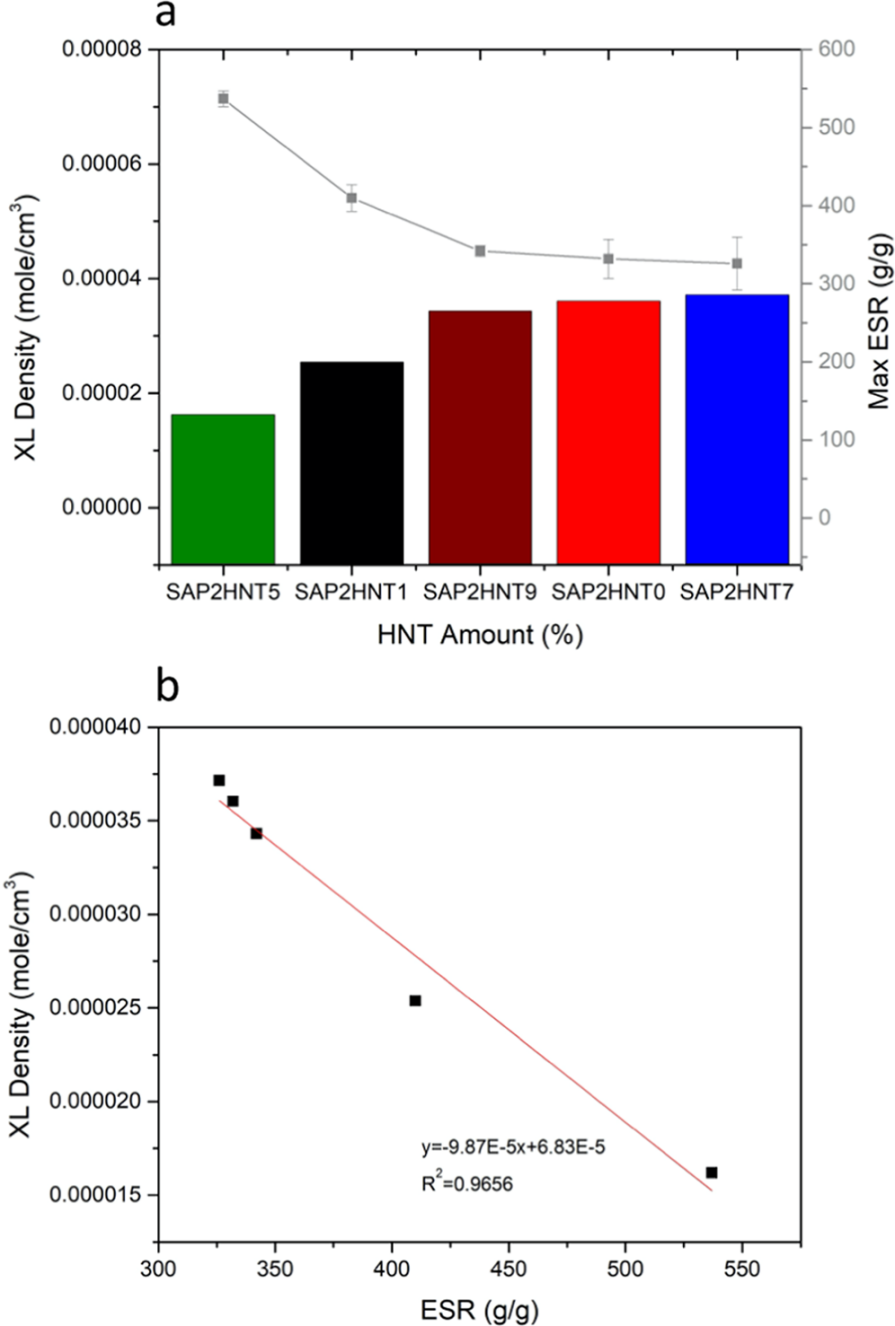
Maximum swelling ratios of the SAP2 dataset in water (a) and crosslink density as a function of ESR (b).

The maximum swelling ratios in the SAP dataset with respect to the HNT amounts are represented in Figure 4a. Here, SAP2HNT5 reaches the maximum swelling ratio with a 537 g/g value. Overall, the swelling kinetics of the SAP dataset confirm that the highest swelling value is observed when 5% HNT is incorporated. All the nanocomposite polymers in our dataset swell 300–500 times their dry weights and completely release the water content in their network within 18–30 days.

### 3.5 Crosslink Density

The swelling test is a useful method to determine the crosslink density of network structures. The measured density (ρ_*SAP*_) values are presented in Table 2, and ρ_S_ = 1.0 for our SAP dataset, and *m*_0_ is set as 0.2 g for all swelling experiments. The Hildebrand solubility parameter of SAPs is determined as 1.49 and 0.086 (kJ cm^3^)^0.5^ for water and SAP (set to poly(ethyl acrylate)),^74^ respectively.

Calculating XL density from the swelling experiments, we demonstrate the linear correlation between swelling and the crosslink density. The inverse proportion between XL and ESR reveals the dependence and proposes that although the HNT filler present in the SAP structure alters the maximum swelling capacities of SAPs (Figure 4a), the XL density is still proportional to the molar volume of SAPs in the nanocomposite structure (Figure 4b). This means that swelling is governed by the XL density, which is an intrinsic property of the synthesized SAPs.

### 3.6 Rheological Behavior of the SAPs

**Figure 5:**
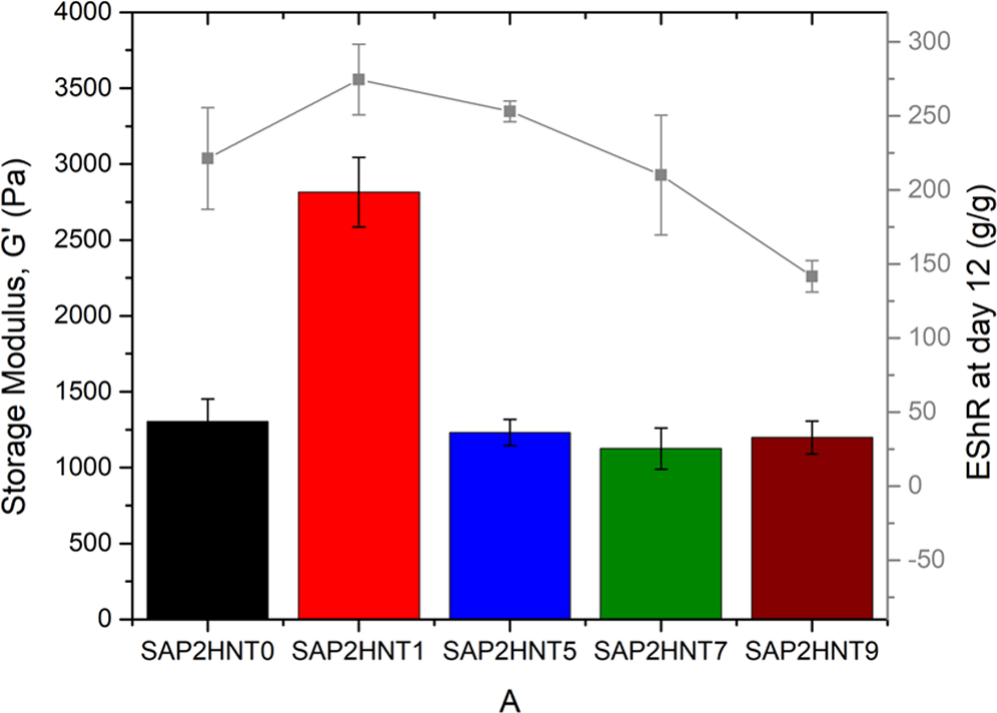
Storage modulus results of SAP and SAP-HNT Nanocomposites.

The comparison of the storage moduli within the SAP dataset reveals that the highest storage modulus is observed in the SAP2HNT1 nanocomposite (Figure 5). This correlates well with water retention profiles where the highest amount of water retention, especially after the 10th day, is observed in SAP2HNT1 (Figure 3b). The storage modulus value decreases in the 5–7% HNT range to values even lower than those of SAP2HNT0 (without HNTs). This can be due to the lack of a homogeneous dispersion of the nanofiller HNTs in the gel matrix, which generates small aggregations confined to the gel matrix. On the other hand, there is a relative increase in the storage modulus values with the increasing HNT amount (9%). Here, the addition of higher amounts of HNTs increases the aggregate diameter and leads to a decrease in the integration between the aggregate and gel matrix.

**Figure 6:**
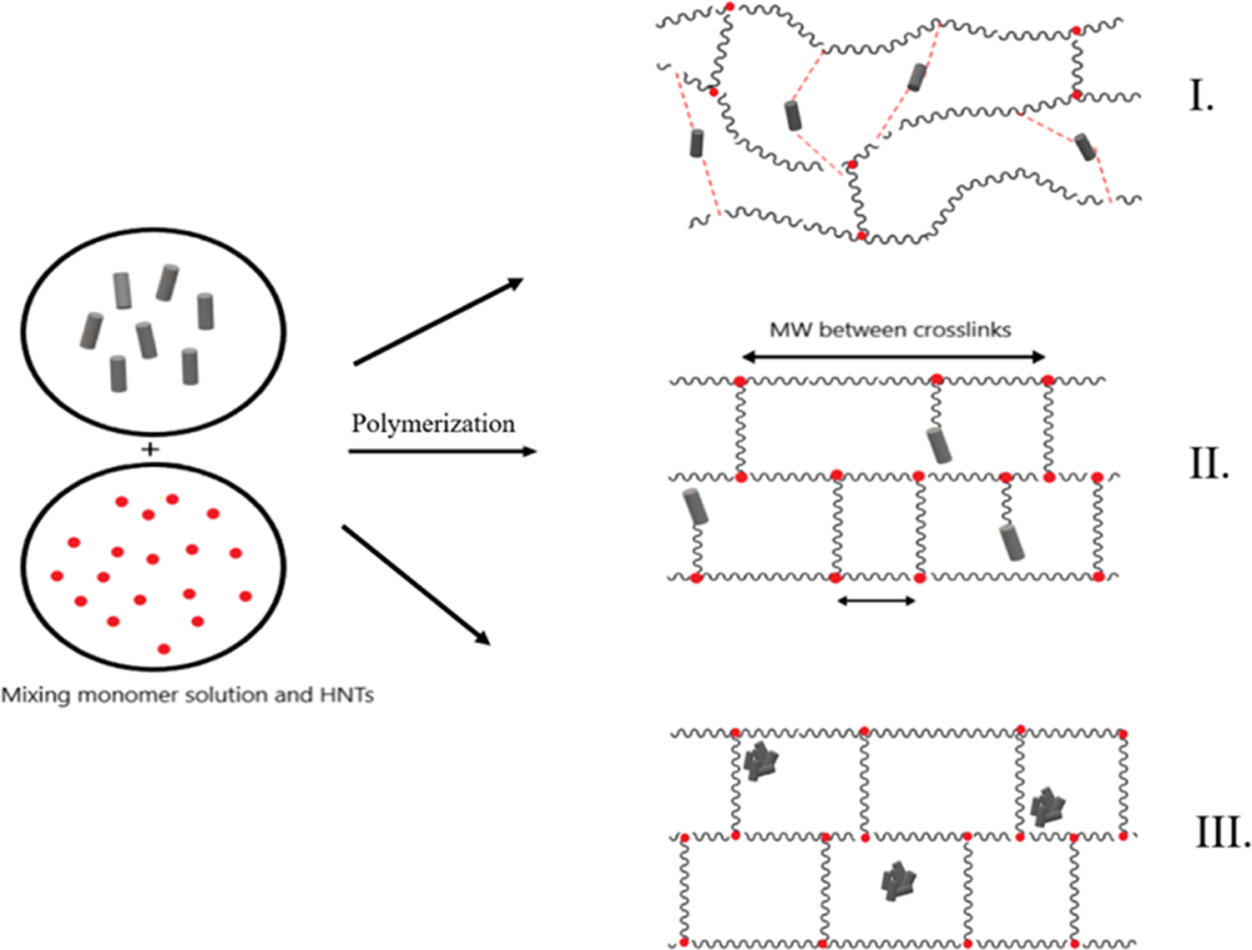
Graphical representation of three different HNT incorporation mechanisms into the SAP nanocomposite structure; intermolecular interactions between the HNT and SAP structure (I), SAP grafting on the HNT surfaces (II), and HNT agglomerated into the SAP structure due to the intramolecular interactions between HNT molecules (III).

As a result, when all rheology measurement results are discussed, it is observed that storage modulus values in the SAP2 dataset have higher values due to the large amounts of the AA monomer in the polymer structure, and the maximum storage modulus value is reached with the addition of 1% HNT. The nonmonotonous trend in *G′* is governed by the three different HNT incorporations into the nanocomposite structure (Figure 6). In the first mechanism, the HNT molecules participate in the nanocomposite structure via secondary interactions. The sharp increase observed in SAP2HNT1 suggests that small amounts of HNTs (around 1 wt %) introduced into the SAP structure results in a positive impact on *G′*. This can be explained by additional intermolecular interactions formed between the HNTs and SAP structure, which may act as physical crosslinking sites and/or entanglement points.^72^ In the second mechanism, as the amount of HNTs introduced into the SAP structure is increased between 1 and 7 wt %, the storage modulus drops because the HNT alters the crosslinking mechanism of the SAP by providing additional binding sites to the silane crosslinker. This gives rise to a competition between self-crosslinking of SAP polymer chains and SAP grafting onto the HNT surface. The more the SAP grafted on the HNT surface, the looser and the less crosslinked the gel becomes, eventually resulting in a lowered storage modulus.^75^ Finally, in the third mechanism, once a certain amount of HNTs is introduced into the nanocomposite structure (>7 wt %), the probability of individual HNT molecules finding each other increases; thus, intramolecular interactions between HNT molecules become the preferred mechanism of interaction for HNTs. This results in HNT agglomerates in the SAP nanocomposite, which gives rise to fewer interfacial interactions between HNT and SAP molecules, eventually fluctuating the storage modulus.^76^ As the gel structures undergo repeated destruction, reconstruction, and agglomeration processes under constant shear,^77^ fluctuating shear responses are observed, particularly after 5% HNT incorporation.

As explained in the first mechanism, the maximum storage modulus was reached in SAP2HN1 with 1% HNT addition due to the physical crosslinking through intermolecular interaction between HNT and SAP structure. The increase in the storage modulus correlates with the water release time of SAP2HN1. Figure 3b presents the longest release period was observed in SAP2HNT1. This correlation was confirmed with the obtained maximum storage modulus with 1% HNT addition and the enhancement in water release time of SAP2HNT1.

## 4 Conclusions

Nanocomposite hydrogels with varying nanofiller contents, high swelling capacities, and longer release times were designed and synthesized by free radical copolymerization of AM, AA, and AMPS in water. The crosslinked and nanocomposite hydrogels were characterized in terms of their structural, thermal, mechanical, and swelling/release properties. It was confirmed from FTIR results that all monomers participated in the polymerization and HNTs are available in the polymer matrix. TGA measurements demonstrate an increase in the thermal degradation temperatures of nanocomposites with respect to the nanofiller addition, while the residual mass increase is directly proportional to the amount of the added HNTs. The highest swelling value is observed in the SAP2HNT5 sample, which has the lowest calculated crosslink density. The rheological characterization of swollen SAPs reaches the maximum storage modulus of 2815 Pa at 25 °C when 1% HNT and an additional amount of the AA monomer were added to the polymer structure. In line with this, as it was found in the swelling/release performance test, the water release time was prolonged up to 27 days with the addition of 1–5% HNT. Among these, the highest water retention profile, particularly after day 10, is observed in SAP2HNT1, which also demonstrates the best storage modulus value. Based on all these results, the maximum swelling ratios of nanocomposite SAPs correlate with the crosslink density of SAPs, while water retention capacities correlate with storage moduli. The rheology investigation suggested a three-phase mechanism of the HNT nanofiller–SAP interaction. In the first phase, nanoparticles are incorporated into the nanocomposite structure via secondary interactions. In the second phase, the additional amount of the nanofiller induced grafting, resulting in a reduced amount of crosslinking. In the third phase, nanofiller agglomerates form, and the modulus responses become fluctuated. A similar nonmonotonous trend is observed in the swelling experiments of SAP-HNT nanocomposites, but this time, the trend is governed by crosslink densities rather than the HNT nanofiller. The current study is a useful explanation of the structure–function relationship for SAP-based nanocomposites and provides insights into the future designs of smart hydrogels.
